# Predictors of Incisional Hernia After Cytoreductive Surgery and HIPEC: A Retrospective Analysis

**DOI:** 10.3390/medicina61081356

**Published:** 2025-07-26

**Authors:** Daniela Di Pietrantonio, Fabrizio D’Acapito, Massimo Framarini, Giorgio Ercolani

**Affiliations:** 1 General and Oncologic Surgery, Morgagni-Pierantoni Hospital, AUSL Romagna, 47121 ForlìItaly, Italy; fabrizioda@gmail.com (F.D.);; 2 Department of Medical and Surgical Sciences (DIMEC), University of Bologna, 40123 Bologna, Italy

**Keywords:** CRS, HIPEC, incisional hernia, eventration, parietal failure, postoperative complications, risk factors

## Abstract

*Background and Objectives*: Incisional hernia is a common complication following cytoreductive surgery (CRS) and hyperthermic intraperitoneal chemotherapy (HIPEC). This study aimed to identify patient and surgical factors associated with its occurrence. *Materials and Methods*: We conducted a retrospective analysis of 122 patients undergoing CRS and HIPEC. Logistic regression models were applied to identify predictors of incisional hernia development. *Results*: Incisional hernia occurred in 23.8% of patients. Hypertension was identified as an independent factor associated with increased risk. Peritoneal Cancer Index (PCI), operative time, and abdominal wall closure technique were not found to be significantly associated with hernia development. *Conclusions:* Preoperative identification of high-risk patients may support the adoption of targeted preventive strategies, including prophylactic mesh placement and enhanced postoperative surveillance.

## 1. Introduction

Cytoreductive surgery (CRS) combined with hyperthermic intraperitoneal chemotherapy (HIPEC) is an established and increasingly utilized treatment modality for primary or secondary peritoneal surface malignancies (PSM), such as mesothelioma, pseudomyxoma peritonei (PMP), and gastrointestinal or ovarian malignancies. This integrated approach has emerged as an essential therapeutic strategy for cancer diseases characterized by predominantly locoregional spread, where systemic chemotherapy alone struggles to achieve effective therapeutic concentrations at the peritoneal level due to the blood–peritoneal barrier. The primary objective of CRS is to achieve complete tumor removal, often necessitating extensive resections of both peritoneal and visceral tissues to effectively reduce tumor burden. After optimal cytoreduction, intraoperative HIPEC is employed to target residual microscopic disease by leveraging the combined effects of hyperthermia and concentrated chemotherapeutic agents. This approach aims to enhance long-term oncological outcomes and increase survival rates.

Although CRS combined with HIPEC carries a considerable risk of short-term complications [1] attributable to the invasiveness of the procedure, its extended operative time, and the physiological effects of hyperthermic chemotherapy, this approach has shown encouraging oncologic outcomes, including enhanced survival rates among appropriately selected patient cohorts [1,2,3,4]. As a result, the procedure’s adoption has increased worldwide, reflecting its potential benefits despite the significant surgical challenges involved.

After a midline laparotomy, one notable long-term postoperative complication is the development of incisional hernia (IH), which can occur in 0–35.6% of patients, according to a meta-analysis by Bosanquet et al. [5]. Incisional hernia (IH) is defined as a defect in the abdominal wall adjacent to postoperative scars, which can be detected either through clinical examination or imaging modalities. Clinical presentations vary from an asymptomatic protrusion to symptomatic cases necessitating urgent intervention, such as those involving incarceration or strangulation. Several factors contribute to the risk of incisional hernia (IH) formation, encompassing both patient-specific characteristics and surgical variables. Established risk factors include obesity, which elevates intra-abdominal pressure on the suture line; history of prior abdominal surgeries that may compromise abdominal wall integrity; female gender; a low suture-to-wound length ratio, suggesting undue tension on the closure; wound infections that hinder proper healing; extended operative duration; increased incision length; and the application of interrupted suturing techniques [5,6,7,8,9].

CRS + HIPEC presents a unique set of intrinsic risk factors for hernia development. The procedure typically requires a large midline incision from the xiphoid process to the pubic area and prolonged operative time, often in patients with a history of prior abdominal surgeries and previous chemotherapy. Chemotherapy, especially when administered intraperitoneally and at elevated temperatures, may impair wound healing due to its direct cytotoxic effects on key reparative cells such as fibroblasts. Additionally, its suppression of the immune system can increase susceptibility to infection, further compromising the healing process. Additionally, surgical removal of abdominal wall components that may be required during cytoreduction can affect the process of achieving closure and maintaining structural integrity [10,11,12]. Despite these considerations, current literature does not demonstrate an increased incidence of incisional hernia with this approach, compared to median laparotomy performed for other indications [13,14,15,16,17,18,19]. Nevertheless, given the significant repercussions of incisional hernia in this already vulnerable patient population, it remains imperative to identify risk factors, enhance postoperative surveillance, and explore preventive strategies aimed at reducing hernia occurrence.

This study is a retrospective analysis of a prospectively maintained database, including 216 consecutive patients who underwent CRS plus HIPEC for PSM. After applying exclusion criteria, 122 patients were included in the analysis. The primary aim of this study is to assess the incidence of incisional hernia following CRS and HIPEC. The secondary objective is to identify potential risk factors associated with IH development in this patient population. Parastomal hernias were not evaluated as part of this study.

## 2. Materials and Methods

This study is a retrospective analysis using data from a prospective database. Although the analysis is retrospective, the data were collected in a standardized manner from the beginning, which minimizes recall bias. The cohort included 216 consecutive patients who underwent CRS with HIPEC for primary or secondary PSM at the General and Oncological Surgery Department of the Morgagni-Pierantoni Hospital in Forlì, Italy, between March 2004 and March 2023. Patients who received a second HIPEC procedure were excluded to maintain cohort uniformity and outcome clarity. The study protocol was approved by Hospital Ethics Committee (Protocol code 0/23453/F2/RP) on 6 May 2004, in accordance with the Declaration of Helsinki (latest revision) and Good Clinical Practice (GCP) guidelines. All patients provided written informed consent prior to surgery.

A total of 94 patients were excluded from the analyses to maintain population homogeneity and ensure reliable data regarding the incidence of incisional hernia. The reasons for exclusion were as follows: 2 due to extensive abdominal wall resection, 5 with pre-existing incisional hernias, 2 who previously had abdominal wall repair with mesh, 23 who required surgery within 24 months due to disease recurrence or bowel obstruction, 23 lost to follow-up, and 39 who died within 24 months postoperatively.

Morbidity was classified using the Clavien–Dindo classification [20].

All procedures were performed via open midline laparotomy and included standardized peritonectomy procedures, organ resections as indicated, and semi-closed-abdomen HIPEC.

A median xipho-umbilical-pubic incision was made. During laparotomy, the extent of peritoneal spread was recorded using the Peritoneal Cancer Index (PCI) [21]. Visceral resection, splenectomy, liver capsule nodule removal, limited liver resection, and other peritonectomy procedures were performed based on distribution of malignancy; healthy peritoneum was preserved as per P.H. Sugarbaker’s method [22]. At the end of cytoreductive surgery, the completeness of cytoreduction score (CCS) was documented according to Sugarbaker’s classification. The CCS assigns a score ranging from 0 to 3: 0 indicates no residual disease; 1 refers to residual disease with a diameter less than 0.25 cm; 2 denotes disease residue between 0.25 and 2.5 cm in diameter; and 3 represents disease residue greater than 2.5 cm in diameter [22].

Following cytoreduction, perfusion was performed using the semi-closed colosseum method [23]. This approach maintains a constant and uniform intraperitoneal temperature and reduces the dispersion of antiblastic-containing vapors into the surrounding environment, which helps protect healthcare personnel. Five drains were placed into the abdominal cavity: three on the left for inflow and two on the right for outflow, all connected with Y-connections to the extracorporeal circulation circuit. The skin of the incision was elevated by Backhaus forceps attached to an autostatic oval retractor anchored to the operating table, creating a cavity suitable for perfusion. The oval was positioned approximately 20 cm above the patient. A polyvinyl chloride sheet covered the central opening, and on top of that sheet was placed the end of the pipe connected to the smoke extractor to minimize the dispersion of antiblastic-containing vapors into the surrounding environment. A volume of approximately 4 L of peritoneal dialysis solution or 5% glucosate solution (equivalent to about 2.2 L/m^2^ of body surface area) was used for circulation, with chemotherapeutic agents—such as mitomycin C, cisplatin, or oxaliplatin—diluted within the solution. The intraperitoneal temperature was maintained at 42–43 °C for either 30 or 60 min, depending on the tumor type.

The midline incision was closed using either a slowly absorbable, single-membrane loop suture in polyglycolic acid with a continuous technique and a suture-to-thread-length ratio of 1:4 or a discontinuous suture made of absorbable synthetic copolymers. In both cases, the distance between stitches and from each stitch to the edge of the incision was 1 cm, and the thread diameter was 1 mm.

Follow-up evaluations were performed one month after surgery to assess the patient’s condition and wound healing. Abdominal and chest CT scans with contrast were scheduled every three months during the first year and every six months during the second year. Clinical evaluations were conducted annually for the first two years. Importantly, the CT scans were not performed under Valsalva’s maneuver, as they were part of routine oncologic follow-up imaging. However, for this study, all CT images were reviewed by an experienced surgeon specializing in abdominal wall surgery, ensuring an accurate and standardized assessment for the identification of IH. IH was defined as a palpable or radiologically confirmed defect in the abdominal wall. Parastomal hernias were not evaluated as part of this study.

### Statistical Analysis

All statistical analyses were performed using Python 3.11 (Statsmodels package), with significance set at *p* < 0.05. Categorical variables were summarized as frequencies and percentages, while continuous variables were expressed as means and standard deviations. To evaluate the association of each variable with the development of incisional hernia, univariate analysis was performed using the chi-squared test or Fisher’s exact test, as appropriate. A multivariate logistic regression analysis was then conducted.

## 3. Results

A total of 122 patients undergoing CRS and HIPEC for PSM were included in the analysis. The median age was 58.7 years, and the mean BMI was 24.4. Most patients were female (71.3%) and non-smokers (67.2%). The median Charlson Comorbidity Index (CCI) was 7.7, and the median PCI was 9.3. Neoadjuvant chemotherapy was administered in 68% of patients. Regarding comorbidities, hypertension was present in 20.5% of patients, chronic obstructive pulmonary disease (COPD) in 4.9%, diabetes mellitus in 6.5%, and obesity (BMI > 30 kg/m^2^) in 9.8%. A previous midline laparotomy was performed in 50.8% of cases. A total of 45% of the patients had ovarian cancer, 27% colorectal cancer, 14% gastric cancer, 10.6% neoplasm of the appendix, 2.4% pseudomyxoma peritonei, and 0.8% mesothelioma. In 41.8%, the muscle fascia was sutured with a continuous suture and in 58.2% with a discontinuous suture. In 61.5% of cases, patients had one or more intestinal anastomoses. Postoperative complications were observed in 19% (*n* = 23) of patients. According to the Clavien–Dindo classification, 9.8% (*n* = 11) of these were classified as major complications (Grade > II). The specific postoperative complications included: one case of anastomotic leak requiring reoperation and terminal colostomy; hemoperitoneum in two patients—one managed conservatively with blood transfusions and the other requiring re-laparotomy for hemostasis; fascial dehiscence in two patients, both necessitating further surgical intervention; intra-abdominal abscesses in three patients, successfully treated with radiologically guided transcutaneous drainage; sub-acute intestinal obstruction in three patients, managed conservatively with gastric tube, fasting, and total parenteral nutrition; pancreatic fistulas in three patients, treated with total parenteral nutrition and continued abdominal drainage; pulmonary embolism in two patients; pleural effusion in two patients, both requiring thoracic drainage; and wound infections in five patients.

Patient characteristics are summarized in Table 1, with early post-operative complications detailed in Table 2.

Within two years post-CRS and HIPEC, 29 patients (23.8%) developed incisional hernia (IH). Of these, twenty were clinically evident, one was detected solely through follow-up CT scan despite being asymptomatic, and eight abdominal wall defects were identified upon review of imaging. Over the 24-month follow-up, only one patient with IH required surgical repair due to severe symptoms.

Univariate analysis identified hypertension as the only variable significantly associated with the development of incisional hernia, with a *p*-value of 0.001 and a positive logistic regression coefficient (logit OR = 1.55), indicating increased risk (see Table 3). This finding was confirmed by multivariate analysis, where hypertension remained the only independent predictor of incisional hernia after CRS + HIPEC (OR: 5.79, 95% CI: 1.82–18.46, *p* = 0.003 (see Table 4).

No other clinical, anesthesiologic, surgical, or postoperative variables—including BMI, CCI grade, abdominal wall resection, closure technique, ostomy, or complications—were statistically significant in the multivariate model.

## 4. Discussion

HIPEC, combined with cytoreductive surgery, is an established treatment for PSM such as pseudomyxoma peritonei, peritoneal mesothelioma, and peritoneal carcinomatosis from colorectal or ovarian cancer [1,2,3]. While this combined approach has improved oncologic outcomes and long-term survival, it is also associated with a high risk of postoperative complications [1,2,3,4], attributable to the extensive nature of the surgery and the biological impact of intraoperative chemotherapy.

Among late postoperative complications, IH is a common concern following abdominal surgery, with reported incidences ranging from 0% to 35.6%, according to a meta-analysis reported by Bosanquet et al. [5]. This systematic review and meta-regression of 14,618 patients from 83 patient groups showed that the prevalence of IH after midline incision was 12.8% (range: 0–35.6%), with a weighted mean of 23.7 months. The estimated risk of undergoing IH repair after median laparotomy was 5.2%. Two meta-regression analyses identified the following characteristics associated with an increased rate of IH: higher age, surgery performed for abdominal aortic aneurysm or obesity surgery, use of a higher median incision, previous laparotomies, and previous IH. In contrast, no significant difference was found in the rate of IH between absorbable and non-absorbable sutures, either alone or in combination with regression analysis.

IH is associated with significant morbidity and diminished quality of life and often necessitates surgical repair, which may become urgent in the presence of complications such as incarceration or strangulation [13,24,25].

The healthcare impact of incisional hernia (IH) is considerable. Patients undergoing cytoreductive surgery (CRS) and hyperthermic intraperitoneal chemotherapy (HIPEC) are especially susceptible to IH due to several factors: previous midline laparotomies that compromise the abdominal wall, the necessity for extensive surgical incisions increasing the risk of dehiscence, prolonged operative times, and bowel edema induced by hyperthermia, which may complicate low-tension abdominal closure and elevate intra-abdominal pressure postoperatively. Additionally, chemotherapy, particularly when delivered intraperitoneally at higher temperatures, can hinder wound healing by exerting cytotoxic effects on tissue-repair cells and suppressing immune function, thereby increasing susceptibility to surgical site infections [1,10,11,12,13,26,27,28]. Surgical resection of involved abdominal wall components during cytoreduction may further compromise closure and structural integrity [4,10,11,12].

Despite these considerations, current literature does not demonstrate a higher incidence rate of IH compared to median laparotomy performed for other surgical procedures. Ray et al. [14] reported an IH rate of 6.9%, identifying obesity, early postoperative complications, neoadjuvant chemotherapy, and bowel anastomosis as significant risk factors. Similarly, Struller et al. [15] reported a 7% IH rate, with age, cardiac comorbidities, and diagnoses of mesothelioma or pseudomyxoma being significant predictors. Ravn et al. [13] found an incidence of 10% after 60 months of follow-up, with older age and fascial dehiscence associated with increased IH risk. Conversely, Ben-Yaacov et al. [16] observed a markedly higher incidence (26.9%) within 6 months, finding a higher American Society of Anesthesiologists (ASA) score (OR 3.9, *p* = 0.012), increased age (OR 1.06, *p* = 0.004), and increased BMI (OR 1.1, *p* = 0.006) to be risk factors in multivariate analysis. Lewcun et al. [17], on the other hand, reported an incidence of 29% over 24 months. The incidence of IH was similar across categories of body mass index, smoking status, and operative time, but they reported that the use of a 4:1 suture length-to-wound length ratio (SL:WL) during fascial closure may reduce IH rates in the HIPEC population; however, they still concluded that larger sample sizes and longer follow-up are needed to determine the statistical significance of this intervention. Finally. Tuttle et al. [18] found a 17% rate, with older age, female sex, and high BMI as independent predictors. A 2023 meta-analysis [19], including nine studies with 1416 patients that compared CRS plus or minus HIPEC, showed a pooled IH incidence of 11%, with slightly higher rates in HIPEC-treated patients (12% vs. 7%), although this difference was not statistically significant (OR 1.9, 95% CI 0.7–5.2; *p* = 0.21). These rates fall within the expected range for midline laparotomies.

Although IH is a well-recognized long-term complication of abdominal surgery, its occurrence after CRS and HIPEC does not appear to be significantly higher than after standard laparotomy in general.

The relatively low incidence may be due to the overall health profile of these patients, who are typically younger (mean age reported between 48 and 65 years across studies) and often have few additional risk factors aside from their cancer [13,14,15,16,17,18,19]. Another possible explanation for the low incidence of incisional hernia in these patients is that extensive cytoreduction combined with peritoneal chemotherapy infusion can lead to numerous intraperitoneal adhesions and altered intra-abdominal pressures, potentially reducing tension on the abdominal wall [29].

However, studies examining long-term complications following CRS + HIPEC are limited; most are retrospective, conducted at single centers, involve small patient cohorts, and are difficult to compare due to differences in methodology, follow-up duration (ranging from 8 to 38 months), definitions of IH, diagnostic criteria, patient populations, and surgical techniques. Despite these limitations, the severe consequences of IH in this already fragile patient population justify the implementation of preventive strategies. Some studies suggest that prophylactic mesh placement [30] or reinforced closure techniques, such as the Reinforced Tension Line (RTL) method [29], may reduce the incidence of IH. Wenzelbert et al. published a study that included 129 patients with CRS/HIPEC between 2004 and 2019 [29]. Band closure was performed in a 4:1 fashion relative to wound length, with either a 2-0 continuous polydioxanone suture (PDS group) or a 2-0 polypropylene suture preceded by an RTL (RTL group). The RTL suture is typically placed within the condensed linea alba, according to the original description [27]. The suture runs parallel to the incision through the fascia on both sides, beginning and ending at the caudal end, where the ends are initially left untied. The incidence of IH in the PDS group was 11%, compared to 2% in the RTL group. Although significance was not reached, the difference is clinically relevant and suggests an advantage with the RTL suture [29].

Therefore, understanding which patients would benefit most from these preventive measures is crucial.

Our retrospective single-center study aimed to assess the incidence of IH within two years after CRS and HIPEC and to identify associated risk factors. We observed an IH incidence of 23.8%, a figure higher than many prior reports but consistent with the broader variability reported in literature, likely reflecting differences in follow-up duration or patient cohorts. Importantly, hypertension emerged as a significant independent predictor of IH. One possible explanation is that hypertension may damage microcirculation, affecting the function of small blood vessels. This can decrease blood flow to the surgical area and limit the delivery of oxygen and nutrients required for healing. In addition, hypertension can adversely affect collagen production and granulation tissue formation, making the fascial scar less robust. These alterations, together with the intrinsic trauma of the suture, can potentially lead to tissue necrosis and delayed healing of the fascia, predisposing patients to the development of hernia [31,32,33].

In contrast, no significant associations were found for obesity, diabetes, CCI score, smoking, neoadjuvant chemotherapy, prior laparotomies, intestinal anastomosis, surgical site infection, PCI score, CCS score, or fascial closure technique (interrupted vs. continuous suture). These results strongly suggest that hypertensive patients are at a significantly higher risk of developing an incisional hernia, independent of other commonly implicated factors.

Our study has several limitations. The retrospective design, relatively small sample size, and follow-up limited to two years may underestimate the true cumulative incidence. Furthermore, follow-up imaging was performed without Valsalva maneuvers, possibly reducing diagnostic sensitivity for IH. However, surgical procedures were standardized, with all operations performed under the supervision of a senior surgeon, and data were retrieved from a prospective institutional database. Further large-scale, prospective, multicenter studies are needed to better define the incidence and risk factors of IH after CRS and HIPEC and to evaluate the efficacy of targeted preventive measures in this high-risk population.

## 5. Conclusions

This study highlights that IH is a relatively common complication following cytoreductive surgery CRS with HIPEC, with an observed incidence of 23.8% at two years. Among the factors analyzed, hypertension was significantly associated with increased IH risk, whereas obesity, diabetes, CCI score, smoking, intestinal anastomosis, surgical site infections, neoadjuvant chemotherapy, PCI score, CCS score, and fascial closure technique were not. Although the incidence does not appear markedly higher than that observed after standard midline laparotomies, the potential morbidity and impact on quality of life in this complex patient population justify focused attention on prevention. Standardization of closure techniques and the evaluation of prophylactic interventions such as mesh placement or reinforced suturing methods may offer benefits. Given the scarcity of dedicated research and the methodological variability among existing studies, there is a clear need for large-scale, multicenter prospective studies. Such investigations are crucial for accurately determining the true incidence of IH following CRS and HIPEC, validating currently identified risk factors, and distinguishing high-risk patients who may benefit from tailored preventive strategies. These efforts will ultimately contribute to enhanced care and improved quality of life for this sensitive patient population.

## Figures and Tables

**Table 1:** Baseline characteristics of the study population.

Characteristics	*n* = 122
Mean Age +/− std deviation (years)	58.7 +/− 11.3
Mean BMI * +/− std deviation (kg/m^2^)	24.4 +/− 4.5
Mean CCI ** +/− std deviation	7.7 +/− 1.3
Mean PCI ° +/− std deviation	9.3 +/− 8.2
Sex	
Female	87 (71.3%)
Male	35 (28.7%)
Previous Midline Incision	62 (50.8%)
Previous chemotherapy	83 (68%)
Comorbidities	
Hypertension	25 (20.5%)
Diabetes	8 (6.5%)
Obesity (BMI * > 30 kg/m^2^)	12 (9.8%)
Chronic obstructive pulmonary disease	6 (4.9%)
Smoking	40 (32.8%)
Primary malignancy	
Ovarian carcinoma	55 (45%)
Colorectal carcinoma	33 (27%)
Gastric carcinoma	17 (14%)
Appendiceal neoplasm	13 (10.6%)
Pseudomyxoma peritonei	3 (2.4%)
Peritoneal mesothelioma	1 (0.8%)

* Body mass index (kg/m^2^); ° PCI, Peritoneal Cancer Index; ** CCI, Charlson Comorbidity Index.

**Table 2:** Early post-operative complications.

Early Post-Operative Complications	*n* (%)
Anastomotic leak	1 (0.8%)
Bleeding	2 (1.64%)
Burst abdomen	2 (1.64%)
Surgical Site Infection	5 (4%)
Intra-abdominal collection	3 (2.46%)
Subacute intestinal obstruction	3 (2.46%)
Pancreatitis/pancreatic leak	3 (2.46%)
Pleural effusion	2 (1.64%)
Pulmonary embolism	2 (1.64%)
Clavien–Dindo Classification	
0	103 (84%)
I	1 (0.8%)
II	7 (5.74%)
III	9 (7.4%)
IV	2 (1.64%)
V	0

**Table 3:** Univariate analysis of risk factors.

Characteristics	Patients with IH * (*n* = 29)	Patients Without IH * (*n* = 93)	*p*-Value
Mean Age +/− std deviation (years)	62.7+/− 8.4	57.2+/−11.7	0.419
Sex			0.6452
Female	22	65	
Male	7	28	
Mean BMI ° +/− std deviation (kg/m^2^)	25.9 +/− 6.4	23.9 +/− 3.7	0.624
Mean CCI ** +/− std deviation	8.2 +/− 1.4	7.5 +/− 1.3	0.108
Mean PCI °° +/− std deviation	9.2+/− 7.8	9 +/− 7.9	0.591
Previous Midline Incision	12	50	0.315
Previous chemotherapy	21	62	0.387
Comorbidities			
Hypertension	12	13	0.0013
Diabetes	2	8	0.876
Obesity (BMI * > 30 kg/m^2^)	4	8	0.363
Chronic obstructive pulmonary disease	2	4	0.531
Smoking	11	29	0.386
Primary malignancy			
Ovarian carcinoma	16	39	0.283
Colorectal carcinoma	7	26	0.864
Gastric carcinoma	3	14	0.799
Appendiceal neoplasm	3	10	0.732
Pseudomyxoma peritonei	0	3	
Peritoneal mesothelioma	0	1	
Surgical data			
Mean duration (minutes)	622.4 +/− 144.5	638.73 +/− 142.87	0.202
Bowel anastomoses	16	59	0.450
Abdominal wall cloture			0.694
Discontinuous suture	15	56	
Continuous suture	14	37	
Early post-operative complications	7	16	0.368

* IH, incisional hernia, ° body mass index (kg/m^2^), ** CCI, Charlson Comorbidity Index, °° PCI, Peritoneal Cancer Index.

**Table 4:** Multivariate analysis of risk factors.

Variable	OR	CI Lower	CI Upper	*p*-Value
Sex	0.8	0.3	2.09	0.6452
BMI °	1.81	0.5	6.54	0.3634
Previous Midline Incision	0.65	0.28	1.51	0.3156
Previous chemotherapy	1.52	0.59	3.96	0.3875
ASA °°	1.32	0.65	2.68	0.4378
CCI *	2.08	0.63	6.8	0.227
Hypertension	4.73	1.83	12.23	0.0013
COPD **	1.75	0.3	10.1	0.5314
Smoking	1.47	0.61	3.53	0.386
Diabetes	1.14	0.22	5.99	0.8761
PCI °*	1.01	0.61	1.68	0.9599
Mean duration	1.0	1.0	1.0	0.8734
Abdominal Wall Resection	0.71	0.22	2.31	0.5729
Ostomy	1.25	0.47	3.36	0.6578
Abdominal wall cloture	1.18	0.52	2.67	0.6944
Surgical complications	1.07	0.93	1.23	0.3685
Clavien–Dindo Classification	1.05	0.69	1.6	0.8133

° Body mass index (Kg/m^2^), °° ASA American Society of Anesthesiology, * CCI, Charlson Comorbidity Index, ** COPD, chronic obstructive pulmonary disease, °* PCI, Peritoneal Cancer Index.

## Data Availability

The datasets used/analyzed during the current study are available from the corresponding author upon reasonable request.

## Abbreviations

The following abbreviations are used in this manuscript:

CRS	Cytoreductive Surgery
HIPEC	Hyperthermic intraperitoneal chemotherapy
PSM	Peritoneal surface malignancies
IH	Incisional hernia
PCI	Peritoneal Cancer Index
CCS	Completeness of cytoreduction score
COPD	Chronic obstructive pulmonary disease
BMI	Body mass index
CCI	Charlson Comorbidity Index
RTL	Reinforced Tension Line
